# An approach to assess the impact of measures mitigating burden associated with COVID-19 in Central Asia (Kazakhstan, Kyrgyzstan, Mongolia)

**DOI:** 10.1038/s41598-026-58200-y

**Published:** 2026-06-22

**Authors:** Nailya Kozhekenova, Natalya Glushkova, Malika Idayat, Zhansaya Nurgaliyeva, Ainash Oshibayeva, Saltanat Kyrykbayeva, Zhanar Zhagiparova, Gulnaz Nuskabayeva, Anastassiya Miller, Arshat Smasheva, Khorolsuren Lkhagvasuren, Batmanduul Erdenebat, Dorjmyagmar Batbayar, Tolkun Djamangulova, Rakhatbek Aidaraliev, Milena Santric Milicevic

**Affiliations:** 1 Faculty of Medicine and Healthcare, Al-Farabi Kazakh National University, 71 Al-Farabi Avenue, Almaty, 050038 Kazakhstan; 2 Faculty of Medicine, Khoja Akhmet Yassawi International Kazakh-Turkish University, Turkestan, Kazakhstan; 3 Health Research Institute, Al-Farabi Kazakh National University, 71 Al-Farabi Avenue, Almaty, 050038 Kazakhstan; 4 Rectorate for Science and Strategic Development, Khoja Akhmet Yassawi International Kazakh-Turkish University, Turkestan, Kazakhstan; 5 Center for Strategic Development, Khoja Akhmet Yassawi International Kazakh-Turkish University, Turkestan, Kazakhstan; 6 School of Sciences and Humanities, Nazarbayev University, Astana, Kazakhstan; 7 Karaganda University Named After Academician E.A. Buketov, Karaganda, Kazakhstan; 8 Department of Health Policy, School of Public Health, Mongolian National University of Medical Sciences, Ulaanbaatar, Mongolia; 9 Center for Health Policy and Management Research, Institute of Public Health, Mongolian National University of Medical Sciences, Ulaanbaatar, Mongolia; 10 Department of Health Information and Technology, Center for Health Development, Ulaanbaatar, Mongolia; 11 Public Association “Healthy Future”, Bishkek, Kyrgyzstan; 12 Institute of Social Medicine, Faculty of Medicine, University of Belgrade, Belgrade, Serbia; 13 Laboratory for Strengthening Capacity and Performance of Health System and Workforce for Health Equity, Faculty of Medicine, University of Belgrade, Belgrade, Serbia

**Keywords:** COVID-19, Mortality, Age-standardized analysis, Central Asia, Gender, Age, Diseases, Health care, Medical research

## Abstract

The COVID-19 pandemic prompted extraordinary response measures in Central Asia** (**Kazakhstan, Kyrgyzstan, and Mongolia), but mortality outcomes varied. This retrospective population-based ecological study assessed age-standardized COVID-19 mortality rates (ASMRs per 100,000) from January 2020 to December 2021. Monthly COVID-19 deaths and population data by age and sex were obtained from official statistical and health sources. ASMRs were calculated using direct age standardization; monthly trends were examined by country, sex, and age group using joinpoint regression and interpreted in relation to governmental mitigation measures and circulating SARS-CoV-2 variants. Rates were higher in men than in women: in Kazakhstan, 24.8 vs. 12.8 in 2020 and 87.3 vs. 67.6 in 2021, in Kyrgyzstan, 46.6 vs. 25.2 and 40.8 vs. 29.7, respectively; and in Mongolia, 91.5 vs. 73.1 in 2021, with no deaths reported in 2020. In 2021, the gap between men and women narrowed as female ASMR increased fivefold. COVID-19 mortality was observed among infants in Kazakhstan and children ages 0–4 in Mongolia (2021). Rates rose sharply from age 60, especially among men and showed distinct country-specific timing: an early peak in Kyrgyzstan, delayed acceleration in Kazakhstan, and a late rise in Mongolia. These results underscore the need for timely, coordinated, age- and sex-sensitive response measures and targeted protection of vulnerable groups.

**Supplementary Information:**

The online version contains supplementary material available at https://doi.org/10.1038/s41598-026-58200-y.

## Introduction

The profound loss of life brought about by the COVID-19 pandemic^1^ has compelled governments to exercise their powers in a manner that modifies various aspects of the informational, physical, social, and economic environments to safeguard the health and well-being of the population while promoting safer behaviors^2^. In 2025, the World Health Organization (WHO) Member States approved the Pandemic Agreement, designed to guarantee fair access to crucial resources for preventing and preparing for pandemics^3^. This included vaccines, protective equipment and medical care. Consequently, conducting a thorough investigation of mortality effects in the lesser-studied countries provides important data on the effects of public health measures and their significance in mitigating severe consequences.

The significance of legislation and regulatory public health measures during a pandemic can be distorted by assessments of their effectiveness^4,5^. Furthermore, the mortality impact of the pandemic can differ across countries, reflecting people’s compliance with government measures^6–8^. In this context, it has appeared crucial for Central Asia’s pandemic management strategy to account for both global and local circumstances amid related uncertainties^9^.

Central Asia, and in particular countries such as Kazakhstan, Kyrgyzstan and Mongolia, is of particular interest for analyzing the impact of the pandemic due to a combination of geographical proximity (Supplementary Figure S1), similar demographic structures (Supplementary Table S1) and the characteristics of healthcare systems inherited from the Soviet model: mainly public funding, low private sector involvement, limited health financing (2–3% of GDP compared to 8–10% in EU countries), limited medical infrastructure, and unequal access to care between urban and rural areas^10,11^. Despite these similarities, countries in the region have shown differences in the course of the pandemic, the timing of the introduction of restrictive measures, and mortality rates^12,13^.

Despite the availability of global and regional estimates of COVID-19 mortality, Central Asian countries such as Kazakhstan, Kyrgyzstan and Mongolia remain under-represented in analytical models^14–17^. Widely used global and regional models: Institute for Health Metrics and Evaluation^14^, the GitHub repository^15^, Li et al.^18^, Imperial College London^17^, Youyang Gu^19^, and Srivastava and Xu^20^, either excluded these countries or considered them only within aggregated regional groups, which limits the possibilities of comparable cross-country analysis.

According to available data, even the WHO’s official figures may not fully reflect the true scale of the pandemic, particularly in its early stages, because of limitations in the diagnosis and registration of COVID-19-related deaths^21^. Official data recorded 19,071 deaths in Kazakhstan, 2,991 in Kyrgyzstan, and 2,136 in Mongolia^22^, while a recent multicenter study based on years of life lost (YLL) showed higher levels of premature mortality in 2021 and marked variation across countries in Central Asia^12^. These findings confirmed the region’s relevance for comparative analysis, highlighted the need for more comparable approaches to mortality assessment, and served as the baseline input for our study.

Despite the availability of some studies, most are based on excess mortality or years of life lost (YLL) indicators and include countries in the region only as part of broader inter-regional samples^12,23^. To the best of our knowledge, no harmonized cross-country comparison of COVID-19 age-standardized mortality rates (ASMR) in Kazakhstan, Kyrgyzstan, and Mongolia has been reported to date.

Thus, the available evidence underscores the importance of the region for comparative analyses of COVID-19-related mortality, particularly in the context of public health and social measures, and for generating findings relevant to pandemic prevention, preparedness, and response. Therefore, this study aimed to examine the trajectory of the COVID-19 epidemic, focusing on how government-imposed mitigation measures evolved, the combined impact of individual adherence to these measures, and the virus’s characteristics, based on the features of the three Central Asian countries where it spread. This approach can help generate useful recommendations from various stakeholders, promoting a collaborative response to the challenges posed by the pandemic.

## Materials and methods

### Study design, study population, and variables

This study used a retrospective, population-based environmental design to examine variations in age-standardized mortality rates (ASMRs) attributed to COVID-19 across three Central Asian countries: Kazakhstan, Kyrgyzstan, and Mongolia, from January 2020 to December 2021. During the study period, the selected countries, united by geographical proximity, comparable demographic structures, and similar pandemic response tasks, had consistent data on COVID-19 deaths, making cross-country comparisons meaningful. This period includes the initial waves of the pandemic, the main peaks in mortality, and the rollout of vaccination, allowing us to study the dynamics of COVID-19 mortality before the major changes in the practice of registering cases and deaths. Cause of death COVID-19 was recorded according to the WHO recommendations: based on real-time polymerase chain reaction (RT-PCR) – «COVID-19, virus identified» (ICD X code—U07.1). Additionally, Kazakhstan and Kyrgyzstan considered code «U07.2» (COVID-19 diagnosed clinically or epidemiologically, without laboratory confirmation).

Three groups of variables were analyzed: SARS-CoV-2 strains, governmental measures, and ASMR.

Governmental measures of interest in the study include major interventions, such as the introduction and removal of quarantine measures, mass vaccination of the population, and political events.

Data for calculating ASMR from January 2020 to December 2021 included monthly COVID-19 death counts, total, by sex (male, female), and age groups from 0 years, 5-year intervals to 85 + (Supplementary Table S2). These age groups were chosen to maintain comparability across countries, since Kyrgyzstan and Mongolia reported data in five-year intervals up to the 85 years and older group, while Kazakhstan used a different approach: < 1, 1–14, 15–17, and 18–30, followed by ten-year intervals: 31–40, 41–50, 51–60, 61–70, and 71–80; the oldest age group included all persons aged 81 and older (Supplementary Table S2). Indicators based on individual age at death (mean and median age at death) were not calculated, as the source data are only available in aggregate format (age groups).

### Data sources, data collection instruments

Data on governmental response measures and circulating virus variants were collected using a narrative review of peer-reviewed articles addressing COVID-19 control policies in Kazakhstan, Kyrgyzstan, and Mongolia. Relevant events and dates were extracted and harmonized across sources to construct a comparative timeline of interventions (Table 1).

**Table 1:** Timeline of key COVID-19 governmental response measures in Kazakhstan, Kyrgyzstan, and Mongolia (2020–2021).

Event	Date	Description
2020 year		
Initial precautions	January 6	**Mongolia.** Gradual introduction of restrictions on travel and movement of the population in early 2020
Emergency	January 30	The World Health Organization (WHO) declared COVID-19 a global health emergency
	February 12	**Mongolia.** The government declares a state of emergency on 12 February 2020
	March 16 to May 11	**Kazakhstan.** From 16 March to 11 May 2020, a state of emergency was declared in the country
	March 25 to May 11	**Kyrgyzstan.** A two-month nationwide lockdown has been imposed since 25 March 2020 due to the COVID-19 epidemic
Quarantine restrictions	June 1	**Kyrgyzstan.** The government lifted most quarantine restrictions: all types of business activity, such as manufacturing and sales, consumer services, tourism and recreational activities, were resumed
The 1st wave of COVID-19	June to August	**Kazakhstan.** The first wave of COVID-19 lasted from late June to late August 2020, peaking in July
		**Kyrgyzstan.** The peak of the pandemic occurred in July 2020, when the highest incidence rates were recorded. The authorities urgently expanded hospital bed capacity for COVID-19 care, strengthened human resources, and deployed mobile teams to provide home care
Lockdown	July 1 to August 15	**Kyrgyzstan.** From 1 July to 15 August 2020, a partial lockdown was introduced
	From July 5	**Kazakhstan.** The government was forced to impose a second (complete) lockdown
Change in statistics (+ viral pneumonia)	July 16	**Kyrgyzstan.** The Ministry of Health has begun combining statistics on COVID-19 and pneumonia cases
	August 1	**Kazakhstan.** Statistical data were combined – Kazakhstan included viral pneumonia in the list of COVID-19 cases and deaths, including cases of pneumonia with a negative result of RT-PCR, but with clinical and epidemiological signs of infection caused by SARS-CoV-2
The 2nd wave of COVID-19	October 2020 to January 2021	**Kyrgyzstan.** The second wave began in the last ten days of September 2020 and continued until the end of December, with a marked surge in October–November following an intense nationwide election campaign in September and major political events in early October
		**Kazakhstan.** The second spike in the incidence of COVID-19, which peaked in December 2020 and January 2021
2021 year		
Start Vaccination	February 1	**Kazakhstan.** The beginning of mass vaccination against COVID-19 (for adults)
	February 23	**Mongolia.** A vaccination campaign has begun and the government has lifted restrictions in an attempt to promote economic recovery
	March 29	**Kyrgyzstan.** Coronavirus vaccination
The 3rd wave of COVID-19	March to May	**Kazakhstan.** A significant spike in morbidity, which lasted until May 31, 2021. This increase in the incidence was due to the widespread spread of British and South African strains of the virus and is associated with the celebration of a public holiday (Nauryz) in March
	April to August	**Kyrgyzstan.** The third wave of COVID-19 began in the last ten days of April 2021, continued until the end of August, and peaked in mid-July
Measures	April 10–25	**Mongolia.** A lockdown was imposed in the country, resulting in a decrease in the number of new cases
	April 26	**Kazakhstan.** The «Ashyq» (the meaning of “Open” in Kazakh) application has been implemented, QR-based digital pass that assigns COVID-19 risk status (red, yellow, blue, green) to regulate access to public venues
Presidential election	May 24 to June 09	**Mongolia.** The presidential election campaign took place throughout Mongolia, and some mass rallies did take place
The 4th wave of COVID-19	June to October 2021	**Kazakhstan.** The surge is associated with the spread of the Delta strain (Indian) in June, the Eta strain (Nigerian) SARS-CoV-2 in August, the maximum increase was noted in place
Vaccination	July 7	**Mongolia.** 55,2% of the total population fully vaccinated
	November	**Kazakhstan.** Vaccination of adolescents (12–17 years old) and pregnant women has begun^24^. RNA vaccine: Comirnaty (Pfizer-BioNTech). The start of adult revaccination
SARS-CoV-2 types	Mongolia, 2021	**Mongolia.** In June–early July 2021, ~ 97% of sequenced cases were Alpha (B.1.1.7), with only rare Delta (B.1.617.2)–compatible mutations

Data sources for mortality and populations are presented in Supplementary Table S2.

Excel templates were prepared for data and information collection using a uniform methodology to ensure comparability of ASMR.

### ASMRs analysis

We applied the direct method of standardisation using crude mortality rates per 100,000 population and the WHO (2000–2001) World standard population (weights) to calculate ASMR to ensure comparability with previous international studies^25^. The WSP, proposed by M. Segi (1960) and later modified and recommended by the WHO, was used as a reference for age distribution in the population.

A multi-stage methodological approach included data pre-processing and, for each country, calculating annual sex- and age-specific mortality rates per 100,000 population (MR)^26^ and ASMR as follows:

$$Mortality\; rate\left( {MR} \right) = \frac{Number \;of\; deaths\; cases\; in \;Age\; Group}{{Total\; Population \;in \;Age\; Group}} \times 100 000$$

Annual mortality rates were calculated separately for males, females, and both sexes in age categories of each country.

The direct standardization method (per 100,000 population) was used to ensure comparability between countries.

$$Age - s\tan dardized mortality rate\left( {ASMR} \right) = \frac{{\sum \left( {MRi \times Wi} \right)}}{100 000}$$

*MRi* — mortality rate in the i-th age group, *Wi* — the weight of this age group in the standard population.

In Kazakhstan, where age categories are shown in a combined format, the overall weight of the standard population was utilized, determined by adding the weights of all 5-year age segments within the range, thereby preventing bias in the standardization process.

The next step was to investigate the monthly dynamics of ASMR, describing the progress of the COVID-19 pandemic in Kazakhstan, Kyrgyzstan, and Mongolia from January 2020 to December 2021. Namely, the identified cross-country temporal patterns and outbreak peaks were further complemented with the information on COVID-19 variants and the introduction of governmental protective measures.

Monthly trends in COVID-19 ASMR were analyzed using joinpoint regression for each country separately (Kazakhstan, Kyrgyzstan, Mongolia) with the programme Joinpoint Regression Program v5.4 (National Cancer Institute, USA)^27^. The model is based on a logarithmic transformation of the mortality rate:

$${\mathrm{ln}}\left( {\mathrm{y}} \right) = {\mathrm{bx}}$$

x—month number (MonthIndex, from 1 = January 2020 to 24 = December 2021), b—regression coefficient, y—monthly ASMR COVID-19 value (per 100,000 population).

This method identifies time points at which the trend changes significantly and estimates the monthly percentage change (MPC, %/month) for each segment and the average monthly percentage change (AMPC), equivalent to the annual average AAPC, is calculated with weights proportional to the length of the segments.

A linear model with constant variance used the dependent variable ASMR (per 100,000 population), with model selection by Permutation test (significance level α = 0.05, maximum 3 join points). With the corresponding 95% confidence interval (95% CI) for each of the fitted line segments.

For log models providing relative (percentage) changes or growth rates rather than absolute increases, the following transformation (to account for zero values) was used:

$${\mathrm{ln}}\left( {{\mathrm{y}} + 0.1} \right) = {\mathrm{bx}}$$

x—month number (MonthIndex, from 1 = January 2020 to 24 = December 2021), b—regression coefficient. y—monthly ASMR COVID-19 value (per 100,000 population).

This calculation smooths out the influence of large outliers, so the model sees the trend less sharply. The number of joinpoints was selected by Weighted BIC. Monthly percent change (MPC, %/month) was estimated for each model segment, with 95% confidence intervals and p-values.

Junction points were interpreted as time points at which the direction or rate of change in ASMR changed significantly, reflecting possible epidemiological or policy shifts (imposition/removal of quarantine, initiation of vaccination, strain surges, etc.).

Log transformation was used to improve the interpretability of mortality trends by expressing changes on a relative scale, reducing the influence of extreme values, and stabilizing variance. Joinpoint regression offers an objective way to identify inflection points and statistically significant shifts in trends, allowing for a more precise determination of epidemic phases and linking them to interventions and changes in infection spread^28^.

### Ethics statement

The study approval of the Ethics Committee of the Al-Farabi Kazakh National University, Almaty, Kazakhstan, was received (Protocol №IRB-A653, dated 07 September 2023). This was a non-interventional study using official aggregated and anonymized mortality and population data, obtained from partner institutions. Informed consent was not required. All methods were performed in accordance with the relevant guidelines and regulations, including the principles of the Declaration of Helsinki.

## Results

### The age-standardised mortality rate from COVID-19 (100 000)

Analyses of ASMR rates from COVID-19 for 2020 and 2021 in Central Asian countries (Kazakhstan, Kyrgyzstan and Mongolia) showed that in all countries, both by year and age group, ASMR rates were higher among men than women (Supplementary Fig. 2 a–c). At the same time, the proportion of women among registered deaths increased in 2021. In Kazakhstan, it rose from 44.8 to 55.1%; in Kyrgyzstan, from 41.5 to 50.2%; and in Mongolia, it stood at 54.6% in 2021.

The key between-country difference was temporal: Kyrgyzstan experienced its highest mortality levels in 2020, Kazakhstan showed an almost fourfold increase in ASMR in 2021, whereas Mongolia shifted from no recorded COVID-19 deaths in 2020 to the highest overall ASMR in 2021.

The highest levels of ASMR were observed in the older age groups (> 60 years old), while among children and adolescents the rates remained extremely low throughout the study period (Supplementary Fig. S2a–c).

Already in 2020, high values were observed in Kyrgyzstan and Kazakhstan, with higher mortality in men than in women: 8.42 versus 5.12 in Kyrgyzstan (65–69 years), 8.3 versus 4.5 in Kazakhstan (61–70 years). In 2021, ASMR increased significantly in Kazakhstan to 28.4 (3.4 times) in men and 24.6 (5.5 times) in women (61–70 years), and ASMR in Mongolia immediately reached high values: 13.5 in men 65–69 years old and 11.9 in women 70–74 years old.

In the middle age group (40–59-years), ASMR remained higher in men than in women across all countries, but, in 2021, the most unfavorable situation was observed in Kazakhstan (Supplementary Figure S2 a-c), In 2020, male ASMR levels were broadly comparable in Kyrgyzstan (1.33–4.17) and Kazakhstan (1.4–3.6), whereas female values were markedly lower in both countries (0.30–2.31 and 0.8–2.1, respectively). By 2021, between-country differences became more pronounced: Kazakhstan showed the highest male ASMR, reaching 5.51 at ages 41–50 years and 10.93 at ages 51–60 years, compared with 1.17 and 3.1 in Kyrgyzstan and 2.53 and 5.16 in Mongolia.

In 2020, ASMR among children was only recorded in Kyrgyzstan, at 0.11. However, Kazakhstan and Mongolia had relatively high ASMR values in the younger age group in 2021: the highest rates are found among infants in Kazakhstan (0.33 for boys and 0.18 for girls) and among children aged 0–4 in Mongolia (0.56 for boys and 0.26 for girls).

### Sex differences in COVID-19 ASMR

**Fig. 1:**
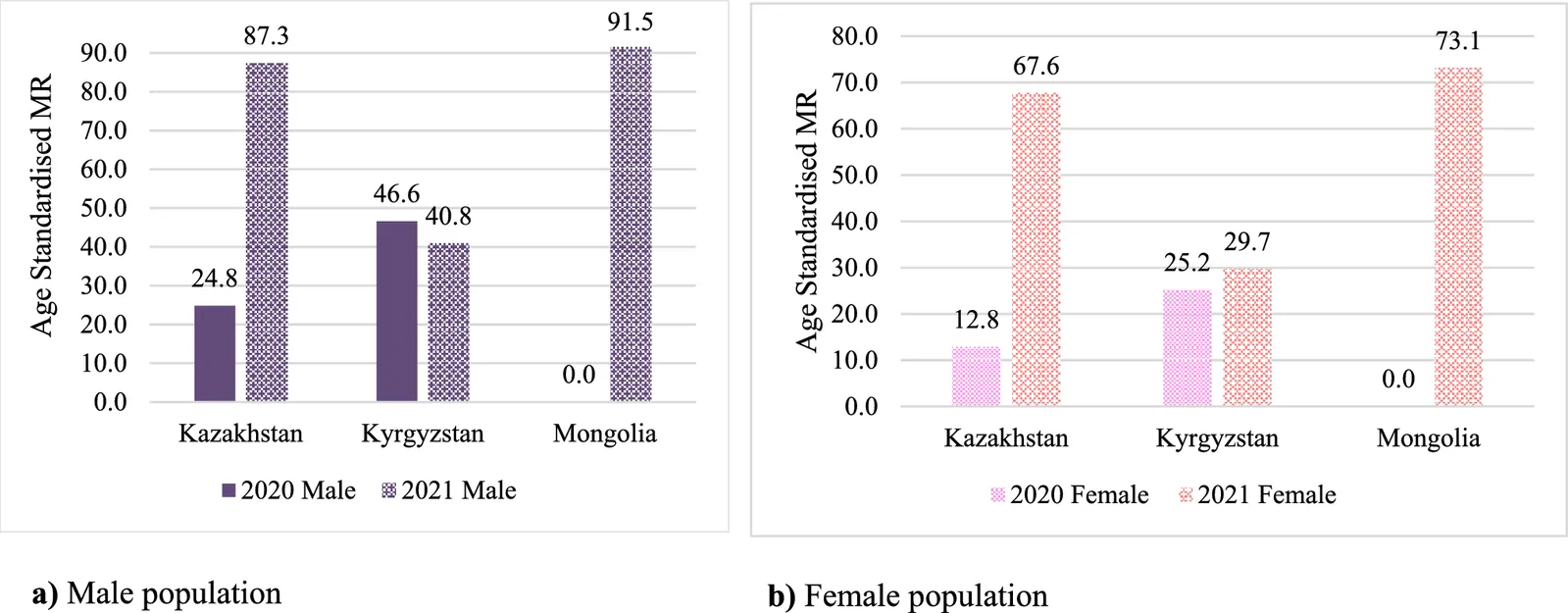
Total age-standardised COVID-19 mortality rates (100 000) by sex (Kazakhstan, Kyrgyzstan, Mongolia), 2020–2021.

The key between-country difference was the pattern of sex-specific ASMR change over time. In 2021, Kazakhstan saw the sharpest rise in mortality rates among women: ASMR for women increased more than fivefold (from 12.8 to 67.6), whereas for men it grew 3.5 times (from 24.8 to 87.3). In Kyrgyzstan, the opposite trend was observed: a moderate increase in mortality among women (by 1.2 times, from 25.16 to 29.6), accompanied by a decrease among men (from 46.6 to 40.8). In Mongolia, COVID-19 mortality was not recorded until 2021, with ASMR reaching 91.5 among males and 73.1 among females, surpassing those of Kazakhstan and Kyrgyzstan during the same period (Fig. 1a, b).

Age-specific analysis of sex differences in ASMR revealed heterogeneity across countries (Supplementary Fig. S2a, b). Although ASMR was consistently higher in men in all settings, the largest male-to-female differences in Kyrgyzstan were observed in working-age adults, reaching 4.5-fold at ages 40–44 years in 2020 and 4.7-fold at ages 35–39 years in 2021. In Kazakhstan, sex differences were more moderate and shifted towards older age groups: from age 31 years onward, male ASMR was 1.6–1.8 times higher than female ASMR, reaching a maximum 2.3-fold difference in those aged over 81 years (Supplementary Figure S3 a, b).

**Fig. 2:**
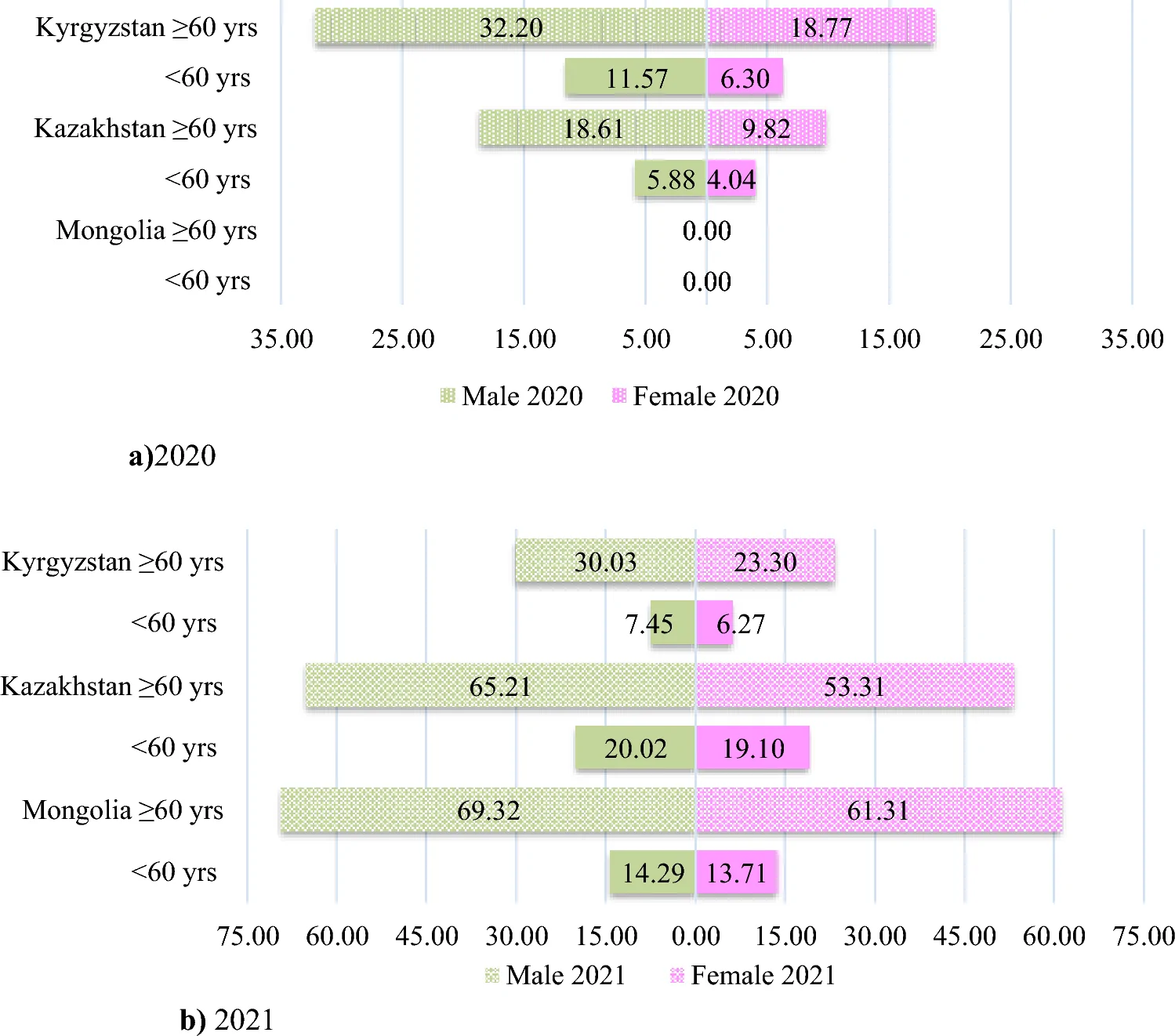
Age standardized mortality rates (100 000) from COVID-19 in Central Asia by year (2020, 2021) and age groups (< 60 years, ≥ 60 years).

When comparing the two broad age groups (< 60 and ≥ 60 years), a marked age gradient persisted in all countries. In 2020, individuals aged 60 and older had ASMR more than double that of those under 60. Men had an ASMR twice as high as women, except for those under 60 in Kazakhstan. In 2021, older men’s ASMR was over three times higher than younger men’s, but rates were similar for both sexes (Fig. 2 a, b).

### COVID-19 (ASMR) burden trends in Central Asian countries

**Fig. 3:**
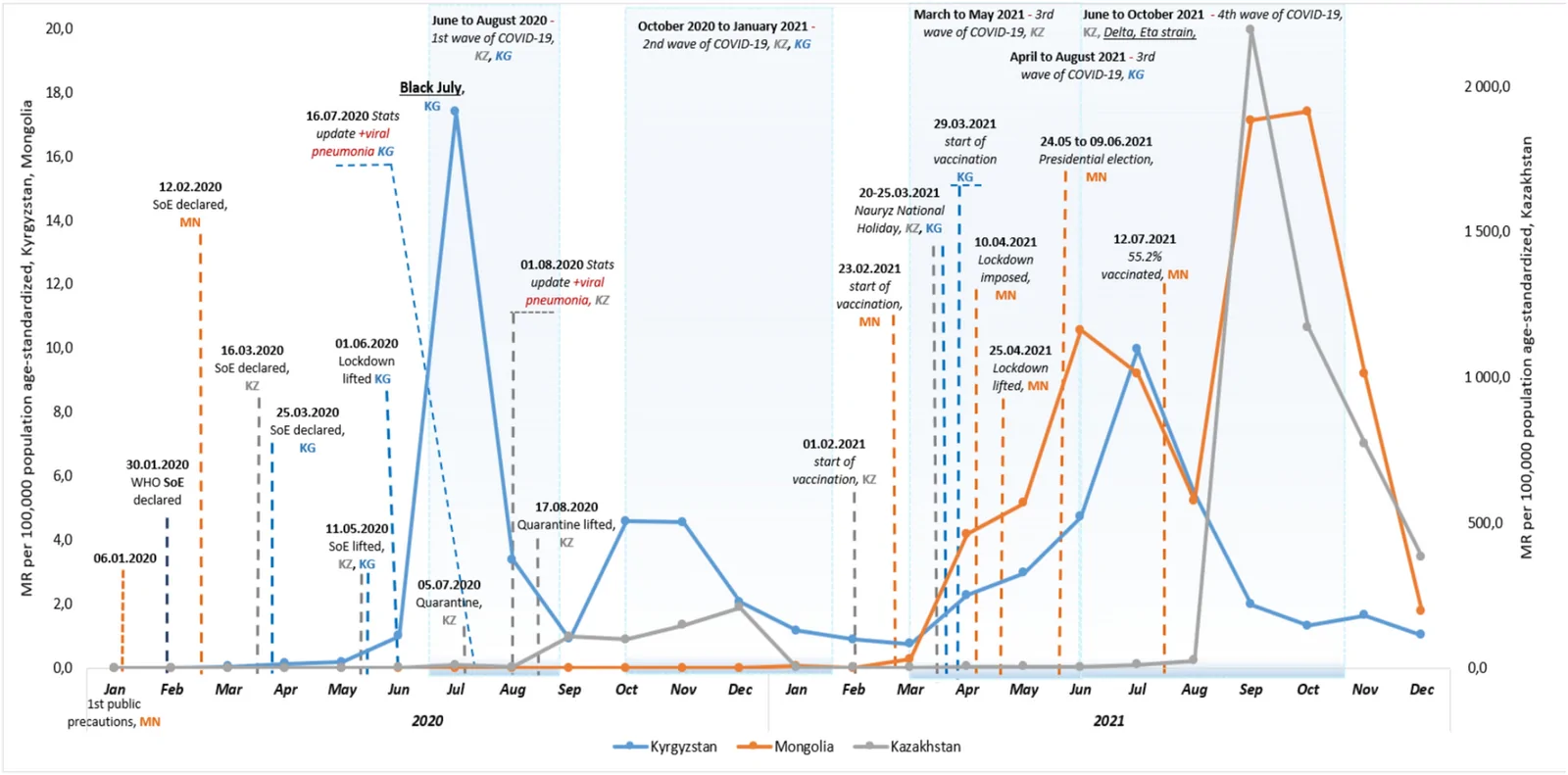
Age-standardized mortality rates from COVID-19 in Central Asia by year (2020, 2021).

Temporal analysis ASMR due to COVID-19 revealed pronounced heterogeneity in both the timing and magnitude of epidemic waves across Central Asian countries (Fig. 3).

In Kyrgyzstan, after the lifting of quarantine measures in June 2020 and the inclusion of pneumonia cases in official statistics, there was a sharp increase in ASMR, culminating in July 2020, at 17.4 per 100,000. Compared with June, ASMR from COVID-19 increased more than threefold in the subsequent month (3.4 times), after which, against the backdrop of the partial lockdown in July–August 2020, a rapid decline in the indicator was observed. The genetic lineage of SARS-CoV-2 circulating in Kyrgyzstan in July 2020 has not been precisely determined, but it is most likely that the early pre-Alpha (Wuhan) variant of the virus was dominant.

In Kazakhstan, the introduction and subsequent adjustment of restrictive measures in 2020 were followed by a delayed but pronounced rise in ASMR during the autumn–winter period. Between August and September 2020, ASMR increased 27.8 times, suggesting a substantial amplification of mortality after earlier policy changes. In 2021, the start of vaccination in February–March was initially associated with low ASMR levels; however, during the Delta-variant period, mortality increased sharply, reaching a peak in September 2021 (2,193.0 per 100,000), corresponding to a 48-fold increase compared with August.

In Mongolia, where restrictive measures were implemented early and COVID-19 mortality was absent in 2020, ASMR began to rise only in 2021. Following the start of vaccination in February 2021, ASMR increased gradually. A moderate summer peak was observed in June, with ASMR 1.8 times higher than in May. A more pronounced increase occurred in autumn 2021, when ASMR rose 3.4 times compared with August. In June and early July 2021, Alpha prevailed among the sequenced cases, while Delta–compatible mutations were recorded only once.

### COVID-19 burden trend analysis (ASMR) in Central Asian countries (ITS analysis)

 Joinpoint analysis of monthly ASMR COVID-19 in Central Asian countries (Kazakhstan, Kyrgyzstan, Mongolia) in the first two years of the pandemic (2020–2021) also showed heterogeneous dynamics across countries (Table 2).

**Table 2:** Joinpoint regression analysis of age-standardized COVID-19 mortality rates per 100 000 in Central Asia (Kazakhstan, Kyrgyzstan, Mongolia), 2020–2021.

Country	Model	Time-series segment	Trend endpoints (MI)	Endpoints (calendar)	Joinpoint(s)	Estimate	95% CI	p-value
Kazakhstan	Linear model	–	–	Full period	–	36.14	10.26; 62.01	0.012*
	Logarithmic linear sensitivity	Full period	1–24	2020–01–2021–12	–	62.76%	− 2.95; 172.96	0.064
		Year 2021	13–24	2021–01–2021–12	–	67.82%	9.30; 157.60	0.018*
	Monthly percent change	Segment 1	1–11	2020–01–2020–11	Nov 2020	151.70%	92.98; 339.33	0.007*
		Segment 2	11–14	2020–11–2021–02	Feb 2021	− 84.45%	− 93.15; − 11.28	0.030*
		Segment 3	14–24	2021–02–2021–12	–	112.88%	55.43; 582.02	0.003*
Kyrgyzstan	Linear model	–	–	Full period	–	0.063	− 0.164; 0.291	0.591
	Logarithmic linear sensitivity	Full period	1–24	2020–01–2021–12	–	18.47%	7.39; 30.70	0.001*
		Year 2021	13–24	2021–01–2021–12	–	− 1.87%	− 8.67; 5.40	0.590
	Monthly percent change	Segment 1	1–7	2020–01–2020–07	Jul 2020	102.01%	37.23; 461.25	< 0.000001*
		Segment 2	7–24	2020–07–2021–12	–	− 1.87%	− 16.61; 6.81	0.617
Mongolia	Linear model	Segment 1	–	–		0.046	− 0.210; 0.303	0.728
		Segment 2	15	2021–03		2.292	1.367; 3.217	< 0.001*
		Segment 3	22	2021–10		− 7.34	− 12.81; − 1.87	0.018*
	Logarithmic linear sensitivity	Full period	1–24	2020–01–2021–12	–	15.07%	0.58; 31.66	0.041*
		Year 2021	13–24	2021–01–2021–12	–	31.05%	− 0.69; 72.90	0.056
	Monthly percent change	Segment 1	1–14	2020–01–2021–02	–	2.15%	− 3.39; 7.17	0.395
		Segment 2	14–17	2021–02–2021–05	May 2021	292.02%	134.77; 368.11	0.022*
		Segment 3	17–22	2021–05–2021–10	–	17.01%	− 2.03; 69.65	0.070
		Segment 4	22–24	2021–10–2021–12	Oct 2021	− 61.92%	− 76.27; − 30.68	0.0004*

*Statistical significance (p < 0.05); MI Month index. MPC Monthly percent change; CI – confidence interval.

**Fig. 4:**
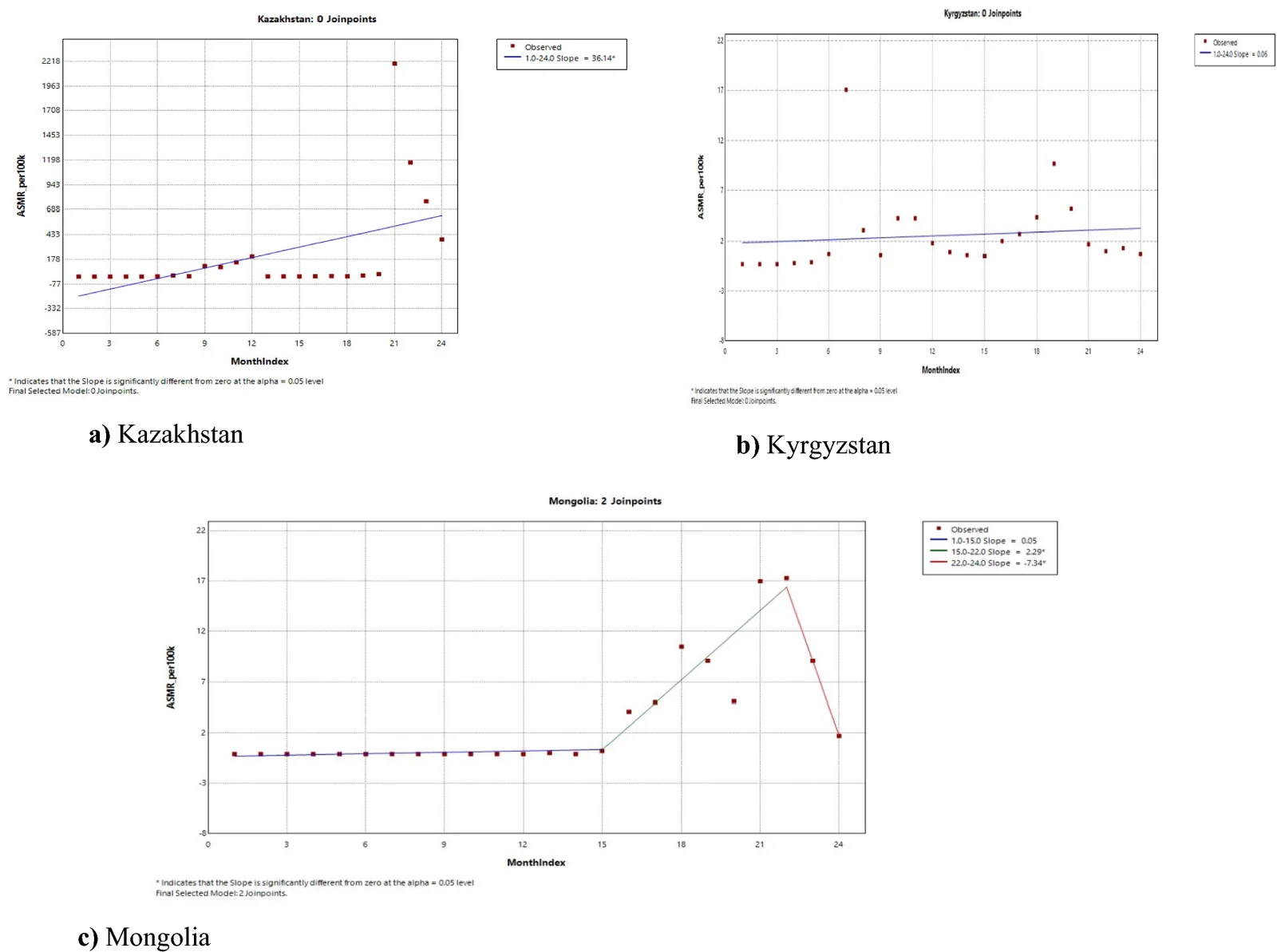
Segmented Joinpoint regression models showing monthly trends in COVID-19 ASMR in Central Asia, 2020–2021.

The Joinpoint linear model for Kazakhstan showed an overall upward trend in ASMR COVID-19, characterized by a significant increase of 36.14 per 100,000 per month (p = 0.012, 95% CI 10.3–62.0). In contrast, in the log-linear sensitivity analysis for the full 2020–2021 period, the positive average monthly percent change (AMPC) in COVID-19 ASMR (+ 62.76%) did not reach statistical significance (95% CI − 2.95 to 172.96; p = 0.064) (Table 2, Fig. 4a).

Sensitive log-rank analysis clarified that this increase in ASMR COVID-19 was not stable over the entire period, but was particularly pronounced and statistically significant, specifically in 2021, 67.82% per month (95% CI 9.30–157.6; p = 0.018) (Table 2).

The ASMR COVID-19 trend in Kazakhstan was characterized by two breaks—November 2020 and February 2021 (Table 2). Until November 2020, there was an increase in ASMR COVID-19 monthly by 151.7 (p = 0.007), declined between November 2020 and February 2021 (MPC − 84.45%; 95% CI − 93.15 to − 11.28; *p* < 0.05), and then increased again from February to December 2021(MPC + 112.88%; 95% CI 55.43 to 582.02; *p* = 0.003).

The slight increase in November coincided with the introduction of improved surveillance, which changed the recording system (1 August 2020) and included «viral pneumonia» in the statistics. The increase continued during the introduction of stringent summer measures (quarantine from 05.07.2020), and their subsequent relaxation on 17.08.2020, but a weekend lockdown was kept. The decline likely reflects the attenuation of the second wave (October–December) with an expected time lag in mortality and the stabilization of the burden on the healthcare system after the autumn peak (Fig. 3). This almost coincides with the start of vaccination (01.02.2021) and the end of the second wave of COVID-19 incidence. In 2021, Kazakhstan relied on regional restrictions, universal mask-wearing, digital entry control (Ashyq), continuous testing and contact tracing, and launched a vaccination campaign. The increase since February is superimposed on the spring third wave associated with the spread of the British and South African variants of the virus after the March mass events (Nauryz) and especially the main rise came in the summer-autumn of 2021 when the Delta variant circulates (Fig. 3).

Kyrgyzstan showed a different pattern. For Kyrgyzstan, the linear model showed no significant overall trend in ASMR (0.063; p = 0.591) (Table 2, Fig. 4b). However,

segment analysis pointed to a single trend reversal in July 2020, at 102.01% (p < 0.000001), coinciding with the peak in mortality and the first wave (Table 2). The growth in ASMR occurred against the backdrop of the lifting of the state of emergency (10 May, transition from emergency mode to quarantine management due to public pressure), the introduction of quarantine (1 June), and accounting changes (16 June, including “viral pneumonia”) (Fig. 3). After reaching its peak, the mortality curve began to decline, probably as the healthcare system stabilized, and the overall incidence of the disease declined by mid-August (Table 2).

In 2021, despite vaccination (since 29 March 2021) and the emergence of the Delta variant in the region, the dynamics remained stable, which was consistent with the absence of a significant average monthly percentage change in 2021 (p < 0.05) (Table 2). Thus, unlike Kazakhstan, the main burden of mortality in Kyrgyzstan was concentrated in 2020, while in 2021, the dynamics remained stable.

Mongolia saw the most delayed trend in ASMR COVID-19. The overall annual AAPC for Mongolia could not be estimated in the primary log-linear joinpoint model because the ASMR was 0 in 2020, rendering log transformation infeasible. Consequently, Mongolia was excluded from the annual joinpoint estimation and was addressed in sensitivity analyses where applicable. In Mongolia, there were two notable spikes in COVID-19 cases in 2021 — in March and October (Table 2, Fig. 4).

The sensitive logarithmic model confirms that, on average, the burden of ASMR COVID-19 increased by 15.07% (p = 0.04, 95% CI: 0.58–31.6) each month over the entire 2-year period (Table 2). However, the average growth rate in 2021 was statistically marginal (+ 31.05%; p = 0.056) due to a sharp subsequent decline after October.

Segment analysis of Joinpoint regression revealed three turning points in the ASMR COVID-19 trend in Mongolia in 2021: February (MI 14), May (MI 17), and October (MI 22). Until February 2021, the trend reflects the effects of early response and strict restrictions (introduction of the first precautionary measures for the population on 6 January 2020, and the state of emergency from February 2020) (Fig. 4c). Since February 2021, ASMR COVID-19 in Mongolia has increased sharply and statistically significantly (MPC =  + 292.0% per month; 95% CI 134.77–368.11; p = 0.022) (Table 2). During this period, the authorities initiated a large-scale vaccination campaign (23 February 2021) and imposed a strict national lockdown from 10 April 2021 (Fig. 3).

The second turning point, in May 2021, was accompanied by moderate but insignificant growth (MPC =  + 17.0%; 95% CI − 2.03–69.65; p = 0.070), coinciding with the lifting of lockdown (25 April) and the election campaign (May–June) amid active vaccination and the spread of the Delta variant. The third turning point, as in all models for Mongolia, reflects a significant monthly decline in ASMR COVID-19 since October (MPC = − 61.9% per month; 95% CI 76.27; − 30.68; p = 0.0004) (Table 2).

## Discussion

Our study of ASMR, stratified by sex and age from January 2020 to December 2021, revealed significant variability in both the timing and severity of COVID-19 mortality across Central Asian countries and different stages of the pandemic, confirming uneven development of the COVID-19 burden in the region. The monthly ASMR showed clear age and gender patterns in COVID-19 mortality: rates were consistently higher among men and increased sharply in age groups 60 years and older. Similar demographic patterns have been reported in regional and international studies using premature mortality indicators, where older age and male gender were considered key factors. The differences observed can be explained by a combination of biological susceptibility, behavioral characteristics, and social determinants of health, and health system differences^29,30^. Cross-country differences should therefore be interpreted cautiously. Although age standardization improved comparability, the observed ASMR patterns may still partly reflect differences in mortality data structure, coding practices, and inclusion criteria for COVID-19 death records, rather than epidemiological variation alone.

A less frequently reported fact in the literature concerns the involvement of younger age groups. In our study, this was particularly pronounced in Kazakhstan and Mongolia in 2021. Although COVID-19 mortality among children remained low in absolute terms, in the youngest age groups it was consistent with data from low- and middle-income countries, where COVID-19-related child mortality, although rare, disproportionately affects infants under 1 year of age and younger children^31,32^. The availability of age-specific data enabled the identification of these subtle yet epidemiologically significant patterns, which are often overlooked in aggregated estimates.

International evidence shows that sex differences in COVID-19 mortality varied by income level and pandemic phase: the male–female mortality gap decreased in several high-income countries in 2021 but remained substantial in middle-income settings^33,34^. The authors attribute the reduction in gender imbalance to the accelerated rollout of vaccination and improvements in clinical management, which may have been particularly beneficial among men, increasing their survival rates. At the macro level, the ratio of male to female deaths was also higher in poorer countries, and as prosperity and urbanization increased, the gap narrowed, with urbanization being associated with a lower mortality ratio^35^.

Explaining the differences requires consideration of the combined influence of the factors of sex and gender^35^. Reviews emphasized the role of differences in immune response and inflammatory reactions, higher prevalence of comorbidity in men, as well as social and behavioral determinants (exposure, risk behaviour, treatment-seeking behaviour (women were more likely to wear masks/practice hygiene/isolate and were more likely to be vaccinated), and barriers to accessing care)^36–38^.

In Central Asian countries, classified as middle-income countries^39^, the analysis by age and gender revealed a similar trend towards a reduction in the relative gender gap within the ASMR in 2021, albeit slower and less pronounced, which probably reflected the delay in vaccination and lower coverage (up to 50% by the end of 2021)^31^. This pattern may indicate unequal and delayed protection across population groups, particularly where vaccination coverage and access to care were uneven^36,37,40^.

In Kyrgyzstan, gender differences were most noticeable among the working-age population, where male mortality significantly exceeded female mortality. In Kazakhstan and Mongolia, gender differences were mainly concentrated among the elderly population. This is consistent with the fact that the contribution of gender factors (employment/exposure structure, treatment models, and barriers to access) may be more critical in working-age groups, whereas comorbidity and clinical vulnerability dominate in older people, where early prevention and access to effective treatment are crucial^36,37^.

Key turning points in the dynamics of ASMR from COVID-19 in Central Asian countries included a sharp peak in the summer of 2020 in Kyrgyzstan, a transition to steady mortality growth in Kazakhstan in early 2021, and a delayed but intense rise followed by a decline in Mongolia in 2021. These patterns indicate that COVID-19 mortality did not follow a single regional trajectory. However, the ecological design of this study does not allow these temporal changes to be attributed directly to specific public health interventions. The identified turning points should instead be interpreted as time markers that may reflect the combined influence of epidemic waves, public health measures, changes in surveillance and reporting, health system capacity, and broader contextual factors.

Despite the almost simultaneous introduction of restrictive measures in Kazakhstan and Kyrgyzstan in March 2020^23,41^, the rise in epidemic indicators in early summer led to the reintroduction of a full lockdown in Kazakhstan and a partial lockdown in Kyrgyzstan^42^. These measures may have contributed to slowing epidemic growth rather than immediately reducing mortality levels; however, their apparent effect is difficult to separate from changes in testing, reporting, health care access, and the timing of epidemic waves^42^. This interpretation is also consistent with evidence from post-Soviet health systems, where the COVID-19 pandemic exposed pre-existing problems, including limited hospital resilience, health workforce constraints, chronic underfinancing, and insufficient preparedness for public health emergencies^13^. In Kazakhstan, this coincided with hospital overcrowding and changes to the registration system^13,43^. In Kyrgyzstan, the summer peak of 2020 (“Black July”) was accompanied by the collapse of the healthcare system and record mortality rates amid the easing of restrictions and the inclusion of pneumonia cases in COVID-19 statistics^29,44,45^. Therefore, the ASMR dynamics in Kazakhstan and Kyrgyzstan should be interpreted not only in relation to the timing of restrictive measures, but also in the context of health system resilience, registration practices, and access to timely medical care.

In contrast, Mongolia introduced strict measures as early as January 2020, which prevented deaths during the first year of the pandemic^12^. However, once restrictions were relaxed and more transmissible variants emerged, Mongolia experienced large waves of infection and a substantial cumulative impact of COVID-19 by the end of 2021, despite high vaccination coverage^46–48^. This pattern aligns with global evidence that early non-pharmaceutical interventions (NPIs) are highly effective at delaying epidemic peaks but rarely prevent eventual widespread transmission unless maintained or combined with other long-term strategies^49,50^. The Mongolian trajectory should therefore not be attributed solely to early restrictions. It may also reflect delayed widespread transmission, subsequent relaxation of restrictions, vaccination timing, changes in circulating viral variants, testing capacity, and case ascertainment. National seroepidemiological data showed very low seroprevalence in 2020–early 2021, followed by a rapid increase by late 2021^47^. Health system capacity and geographic barriers related to low population density and nomadic settlement patterns may have further influenced access to timely care^51^. In our analysis, the increase in COVID-19 ASMR in 2021 is compatible the interpretation that early measures delayed, rather than fully prevented, the subsequent rise in COVID-19 mortality.

Despite differences in the timing of the onset of epidemic phases in Central Asian countries, the main increase in ASMR COVID-19 occurred in 2021. This coincided with the period of dominance of the Delta variant, which was characterized by higher transmissibility and an increased risk of severe disease and death compared to previous strains^52^. A similar shift in mortality peaks in 2021 has previously been described for middle-income countries, including India and Indonesia in South Asia, Mexico and Brazil in Latin America, and several countries in the Middle East^12,31,53^, where Delta waves coincided with insufficient and delayed vaccination coverage^23,53^. At the same time, international multilevel models did not reveal a statistically significant association between vaccination coverage and excess mortality in 2021, highlighting marked cross-country heterogeneity^16^. The short-term protective effect of vaccination was mainly seen in high-income countries (Austria, Denmark, Israel, United Kingdom), while in middle-income countries (Georgia, Ukraine), it was often offset by the late start of vaccination and the coincidence with the Delta wave^16^. Structural constraints on health systems and delays in hospitalization, previously associated with increased COVID-19 mortality in middle-income countries, might have played an additional role in shaping the observed mortality burden^23,53^.

In Kazakhstan, the ASMR increase in 2021 coincided with limited vaccination coverage (~ 42%) and circulation of the Delta strain^31,54–56^. In contrast, in Mongolia, higher vaccination coverage (66.5%) combined with anti-epidemic measures was accompanied by a short-term increase in ASMR (February-May) and continued control measures coincided with a shorter ASMR increase followed by a decline^57,58^.

## Implications

From a practical point of view, our results indicate that pandemic preparedness should be assessed not only in terms of the severity of response measures, but also on the timeliness of introduction and lifting individual non-pharmaceutical interventions (NPIs), their targeting, alignment consistency with healthcare system capacity, and the speed of scaling up pharmaceutical protection (vaccination, therapy)^40^. These findings do not imply that individual measures can be evaluated in isolation; rather, they emphasize the need to create integrated monitoring systems based on sex- and age-disaggregated indicators to identify vulnerable subgroups, and adjust response strategies^35,36,38^.

Given the consistently higher mortality rate among men and marked age-specific differences, response strategies should focus on high-risk groups^35,38^. For working-age groups, priorities include reducing exposure workplace prevention, early diagnosis, and lowering barriers to care; for older adults—priority should be given to prevention (vaccination/boosters) and access to effective treatment for decompensated chronic diseases^35–38^. Digital monitoring systems, telemedicine, vaccination registries may strengthen health-system response capacity.

To improve the interpretation of the effectiveness of measures, it is advisable to supplement the analysis with monitoring of key links in the testing-to-outcome chain (including indicators of treatment/testing and gender differences in severe outcomes, such as hospitalization/intensive care)^35,36^. Finally, in cross-country comparisons, it is important to explicitly account for possible differences in cause-of-death registration and testing availability to separate the real effects of policy from accounting artefacts^35^.

The study of standardized COVID-19 mortality in Central Asian countries has not only regional but also international research significance. By providing a country-specific analysis of observed mortality trends in Kazakhstan, Kyrgyzstan, and Mongolia, broken down by age and gender, our study provides empirical data necessary to refine existing models and develop pandemic preparedness strategies tailored to specific conditions. Our analysis went beyond annual aggregate estimates to provide a monthly epidemic trend for each country, reflecting the real-time dynamics of ASMR fluctuations. This approach allows the mortality impact of COVID-19 to be assessed and provides a basis for subsequent analyses of pandemic response policies.

## Limitations

The study faced several challenges, including variations in mortality by the age and sex structure of the population, leading us to use standardized rates for cross-country comparisons. However, during the epidemic, countries report mortality data that reflect differences in official procedures for diagnosis, registration, and classification of causes of death, as well as the pandemic’s impact. The use of the World Standard Population (WHO 2000–2001) may influence the magnitude of mortality estimates, particularly in Central Asian countries with relatively young population age structures. Therefore, for national assessments, specific rates rather than WHO-ASMR are more informative. However, relative comparisons across countries and sexes, as well as temporal trends over the study period, are expected to remain robust with the WHO-ASMR.

Cross-country comparisons may be affected by differences in COVID-19 diagnosis, testing availability, death certification, cause-of-death registration, ICD-10 coding, reporting practices, mortality surveillance, and health-system capacity. Therefore, observed differences in ASMR may reflect both true epidemiological variation and differences in registration or reporting. Broader political, socioeconomic, and governance contexts may also have influenced. The analysis could be further strengthened by including other Central Asian countries, such as Uzbekistan and Tajikistan, if comparable age- and sex-specific mortality data become available.

As an ecological study, it is subject to ecological fallacy, meaning that population-level associations may not reflect individual-level relationships. The use of population-level data limits the ability to assess individual-level responses to interventions and may lead to over- or underestimation in specific subgroups. Thus, these findings may help generate hypotheses for future research, but should not be interpreted as direct evidence of the effectiveness of individual interventions.

## Conclusion

This comparative assessment of age-standardized COVID-19 mortality in Central Asian countries (Kazakhstan, Kyrgyzstan, and Mongolia) (January 2020–December 2021) demonstrates pronounced cross-country heterogeneity in both the timing and magnitude of mortality waves, despite geographic proximity and broadly comparable health-system legacies.

In all countries, mortality increased significantly with age, and ASMR rates were higher among men, indicating a consistent sex-age risk gradient. At the same time, changes in the relative contribution of women to registered deaths were observed in 2021, highlighting the need to interpret gender differences taking into account the age structure and temporal phase of the epidemic.

Importantly, the observed burden was temporally concentrated in distinct phases: Kyrgyzstan experienced an early and sharp peak in 2020 followed by stabilization, Kazakhstan exhibited a delayed but marked acceleration with the main lethal burden shifting to 2021, and Mongolia had no registered COVID-19 mortality in 2020 but showed a delayed, intense rise and subsequent decline in 2021.The identified differences indicate the context-dependent influence of a combination of government measures, their compliance by the population, and the characteristics of circulating variants on the formation of the lethal burden.

These findings highlight the importance of an integrated and adaptive response to pandemics. Strengthening health system capacity, ensuring timely vaccination and access to effective treatment, especially for high-risk groups, and addressing age and gender inequalities are all essential components of an effective strategy. In addition, improving surveillance systems and ensuring accurate and comparable cause-of-death reporting are critical for understanding and responding to future health crises.

Overall, the results emphasize that reducing pandemic mortality depends not only on the presence of interventions, but on their timing, coordination, and ability to address underlying age and gender inequalities.

## Supplementary Information

Supplementary Information.

## Data Availability

The datasets used and analysed during the current study are available from the corresponding author on reasonable reques.

## Abbreviations

COVID-19: Coronavirus disease 2019
ASMR: Age-standardized mortality rates
SARS-CoV-2: Severe Acute Respiratory Syndrome Coronavirus 2
WHO: World Health Organization
GDP: Gross domestic product
EU: European union
YLL: Years of life lost
RT-PCR: Real-time polymerase chain reaction
ICD X: International classification of diseases 10th revision
RNA: Ribonucleic acid vaccine
WSP: World standard population
MR: Mortality rate
MRi: Mortality rate in the i-th age group
Wi: The weight of this age group in the standard population
USA: United States of America
AMPC: Average monthly percentage change
AAPC: Average annual percent change
CI: Confidence interval
BIC: Bayesian information criterion
MPC: Monthly percent change
MI: Month index
NPIs: Non-pharmaceutical interventions
