# Supplementary material for: An approach to assess the impact of measures mitigating burden associated with COVID-19 in Central Asia (Kazakhstan, Kyrgyzstan, Mongolia)
Source: Sci Rep. 2026 Jun 22;16:28641. doi: 10.1038/s41598-026-58200-y (PMC13575138; doi:10.1038/s41598-026-58200-y)
Supplement: Supplementary file 1 [file 41598_2026_58200_MOESM1_ESM.docx]

Supplementary information

Supplementary Table S1. Country profile indicators for Kazakhstan, Kyrgyzstan and Mongolia (2020)

| Indicators | Kazakhstan | Kyrgyzstan | Mongolia |
| --- | --- | --- | --- |
|  | **2020** | | |
| Population / % of female population^1^ | 19 482 117 –  51.48% female | 6 726 596 –  50.5% female | 3 327 204 –  49.9% female |
| average age of the population^2^ | 29.2 | 24.6 | 26.4 |
| % of rural population^2^ | 23.33% | 24.5% | 29.5% |
| Crude death rate^2,3^  (deaths per 1000 population) | 8.6 | 7.7 | 6.5 |
| Three leading causes of death, excluding COVID-19, per 100,000 population^3^  (all ages, both sexes) | Ischemic heart disease – 169.2 | Ischemic heart disease – 148.15 | Ischemic heart disease – 93.30 |
|  | Stroke – 120.07 | Stroke – 59.83 | Stroke – 68.35 |
|  | Cardiomyopathy, myocarditis, endocarditis – 36,76 | Cirrhosis of the liver – 25,16 | Liver cancer – 55,31 |
| GDP per capita, PPP^1^  (current international $) | 29039.8 | 5178.6 | 13693.2 |
| Current health expenditure per capita, PPP^1^  (current international $) | 1089.9 | 258.0 | 646.0 |
| Coverage of social insurance programs^1^  (% of population) | 40.3% | 49.4% | 37.0% |
| Primary completion rate, total^1^  (% of relevant age group) | 97.8% | 98.8% | 95.2% |

Legend: indicators were extracted from

^1^World Bank, World Development Indicators

^2^United Nations, Data Portal Population Division

^3^World Health Organization, Global health estimates

Supplementary Table S2. Sources of data on COVID-19 mortality in Central Asian countries

| **Country** | **Data source**  (mortality and populations) | **Age groups** | **ICD-10 codes** | **Death records** |
| --- | --- | --- | --- | --- |
| Kazakhstan | Ministry of Health of the Republic of Kazakhstan;  Agency for Statistics of the Republic of Kazakhstan | <1;  1–14;  15–17;  18–30;  then the ten-year groups (31–40, …, 71–80);  81 plus | U07.1, U07.2 | It is not specified whether only cases with COVID-19 as the underlying cause of death or also co-morbidities were counted |
| Kyrgyzstan | National Statistical Committee of the Kyrgyz Republic | from 0 years, five-year intervals to 85+ | U07.1, U07.2 | Both deaths with COVID-19 as the underlying cause and deaths with COVID-19 as an associated condition are included |
| Mongolia | Health Development Centre, Ministry of Health of Mongolia | from 0 years, five-year intervals to 85+ | U07.1 | Both deaths with COVID-19 as the underlying cause and deaths with COVID-19 as an associated condition are included |

*Table Source of Information: Modification of original table* [17].

Supplementary Figure S1

Supplementary Figure S1. Geographic location of Kazakhstan, Kyrgyzstan, and Mongolia

Age Standardised MR

|  | 2020 Male | 2020 Female |
| --- | --- | --- |
| 0-1 | 0 | 0 |
| 1 to 14 | 0 | 0 |
| 15 to 17 | 0 | 0 |
| 18 to 30 | 0.102084 | 0.218214 |
| 31 to 40 | 0.410046 | 0.249262 |
| 41 to 50 | 1.39088 | 0.766529 |
| 51 to 60 | 3.57254 | 2.11249 |
| 61 to 70 | 8.32782 | 4.46374 |
| 71 to 80 | 7.52208 | 3.50932 |
| 81 plus | 3.48005 | 1.4894 |

Age Standardised MR

|  | 2021 Male | 2021 Female |
| --- | --- | --- |
| 0-1 | 0.328914 | 0.175931 |
| 1 to 14 | 0.0144788 | 0.0153547 |
| 15 to 17 | 0 | 0.0239583 |
| 18 to 30 | 0.495773 | 0.69602 |
| 31 to 40 | 1.83451 | 1.58008 |
| 41 to 50 | 5.51412 | 4.12308 |
| 51 to 60 | 10.8627 | 9.53612 |
| 61 to 70 | 28.4142 | 24.5844 |
| 71 to 80 | 28.052 | 19.091 |
| 81 plus | 11.8095 | 7.82149 |

a) Kazakhstan

Age Standardised MR

|  | 2020 Male | 2020 Female |
| --- | --- | --- |
| 0–4 | 0.0580741 | 0.0305988 |
| 5–9 | 0 | 0.0270776 |
| 10–14 | 0.0290889 | 0 |
| 15–19 | 0.0347962 | 0.108356 |
| 20–24 | 0.273515 | 0.126623 |
| 25–29 | 0.303638 | 0.114647 |
| 30–34 | 0.25471 | 0.106598 |
| 35–39 | 0.808208 | 0.298839 |
| 40–44 | 1.33322 | 0.29617 |
| 45–49 | 2.59455 | 1.14602 |
| 50–54 | 3.00739 | 1.78915 |
| 55–59 | 4.16818 | 2.30728 |
| 60–64 | 7.69859 | 5.31384 |
| 65–69 | 8.41586 | 5.1243 |
| 70–74 | 7.69994 | 4.36525 |
| 75–79 | 4.99659 | 2.18609 |
| 80–84 | 2.9191 | 1.30645 |
| 85 plus | 2.00931 | 0.51058 |

Age Standardised MR

|  | 2021 Male | 2021 Female |
| --- | --- | --- |
| 0–4 | 0.088115 | 0.0926996 |
| 5–9 | 0 | 0 |
| 10–14 | 0 | 0 |
| 15–19 | 0 | 0.0352177 |
| 20–24 | 0.0625628 | 0.0650459 |
| 25–29 | 0.083791 | 0.174692 |
| 30–34 | 0.229132 | 0.14692 |
| 35–39 | 0.485317 | 0.102749 |
| 40–44 | 1.03447 | 0.642058 |
| 45–49 | 1.17044 | 1.13425 |
| 50–54 | 1.95954 | 1.63706 |
| 55–59 | 3.09989 | 2.34107 |
| 60–64 | 4.19902 | 3.76932 |
| 65–69 | 8.93753 | 6.17364 |
| 70–74 | 7.49952 | 4.9017 |
| 75–79 | 5.19072 | 4.1616 |
| 80–84 | 3.87291 | 2.10776 |
| 85 plus | 2.91918 | 2.17929 |

b) Kyrgyzstan

Age Standardised MR

|  | 2020 Male | 2020 Female |
| --- | --- | --- |
| 0–4 | 0 | 0 |
| 5–9 | 0 | 0 |
| 10–14 | 0 | 0 |
| 15–19 | 0 | 0 |
| 20–24 | 0 | 0 |
| 25–29 | 0 | 0 |
| 30–34 | 0 | 0 |
| 35–39 | 0 | 0 |
| 40–44 | 0 | 0 |
| 45–49 | 0 | 0 |
| 50–54 | 0 | 0 |
| 55–59 | 0 | 0 |
| 60–64 | 0 | 0 |
| 65–69 | 0 | 0 |
| 70–74 | 0 | 0 |
| 75–79 | 0 | 0 |
| 80–84 | 0 | 0 |
| 85 plus | 0 | 0 |

Age Standardised MR

|  | 2021 Male | 2021 Female |
| --- | --- | --- |
| 0–4 | 0.558827 | 0.261994 |
| 5–9 | 0 | 0.104833 |
| 10–14 | 0.111181 | 0.173716 |
| 15–19 | 0.0816719 | 0.169907 |
| 20–24 | 0.142581 | 0 |
| 25–29 | 0.134694 | 0.135967 |
| 30–34 | 0.368432 | 0.369471 |
| 35–39 | 0.889527 | 0.695803 |
| 40–44 | 1.30982 | 1.06457 |
| 45–49 | 2.53167 | 1.6236 |
| 50–54 | 3.05072 | 2.84668 |
| 55–59 | 5.16397 | 4.91645 |
| 60–64 | 9.82572 | 9.47913 |
| 65–69 | 13.527 | 11.6385 |
| 70–74 | 18.4023 | 11.8979 |
| 75–79 | 12.3563 | 9.79935 |
| 80–84 | 9.25285 | 7.23356 |
| 85 plus | 13.7943 | 10.6663 |

c) Mongolia

Supplementary Figure S2. Assessment of COVID-19 age-standardized mortality in Central Asia by age and gender for 2020-2021, 100 000

a) Kyrgyzstan and Mongolia

Rate ratio (RR)

|  | Kyrgyzstan 2020 | Kyrgyzstan 2021 | Mongolia 2021 |
| --- | --- | --- | --- |
| 0–4 | 1.89792 | 0.950543 | 2.13297 |
| 5–9 | 0 | 0 | 0 |
| 10–14 |  |  | 0.640016 |
| 15–19 | 0.321128 | 0 | 0.480685 |
| 20–24 | 2.16007 | 0.961825 |  |
| 25–29 | 2.64845 | 0.479649 | 0.990639 |
| 30–34 | 2.38945 | 1.55957 | 0.997189 |
| 35–39 | 2.7045 | 4.72331 | 1.27842 |
| 40–44 | 4.50155 | 1.61117 | 1.23038 |
| 45–49 | 2.26397 | 1.03191 | 1.5593 |
| 50–54 | 1.68091 | 1.19699 | 1.07167 |
| 55–59 | 1.80653 | 1.32413 | 1.05035 |
| 60–64 | 1.44878 | 1.114 | 1.03656 |
| 65–69 | 1.64234 | 1.44769 | 1.16226 |
| 70–74 | 1.76392 | 1.52998 | 1.54668 |
| 75–79 | 2.28563 | 1.24729 | 1.26093 |
| 80–84 | 2.23437 | 1.83745 | 1.27916 |
| 85 plus | 3.93534 | 1.33951 | 1.29326 |

b) Kazakhstan

Rate ratio (RR)

|  | Kazakhstan 2020 | Kazakhstan 2021 |
| --- | --- | --- |
| 0-1 |  | 1.86957 |
| 1-14 |  | 0.942952 |
| 15-17 |  | 0 |
| 18-30 | 0.467816 | 0.712296 |
| 31-40 | 1.64504 | 1.16102 |
| 41-50 | 1.81452 | 1.33738 |
| 51-60 | 1.69115 | 1.13911 |
| 61-70 | 1.86566 | 1.15578 |
| 71-80 | 2.14346 | 1.46938 |
| 81 plus | 2.33655 | 1.50988 |

Supplementary Figure S3. Male-to-female rate ratios of age-standardized COVID-19 mortality by age group
